# Genetic characterization and phylogenetic analysis of porcine epidemic diarrhea virus in Guangdong, China, between 2018 and 2019

**DOI:** 10.1371/journal.pone.0253622

**Published:** 2021-06-24

**Authors:** Feng Wen, Jing Yang, Anqi Li, Zhonggui Gong, Lulu Yang, Qing Cheng, Congying Wang, Mengmeng Zhao, Sheng Yuan, Yao Chen, Saeed El-Ashram, Yong Li, Hai Yu, Jinyue Guo, Shujian Huang

**Affiliations:** 1 College of Life Science and Engineering, Foshan University, Foshan, Guangdong, China; 2 Center for Animal Disease Control and Prevention, Shaoguan, Guangdong, China; 3 College of Animal Science and Technology, Jiangxi Agricultural University, Nanchang, Jiangxi, China; 4 Shanghai Veterinary Research Institute, Chinese Academy of Agricultural Sciences, Shanghai, China; Taif University, SAUDI ARABIA

## Abstract

Porcine epidemic diarrhea virus (*PEDV*), a leading cause of piglet diarrhea outbreaks, poses a significant danger to the swine industry. The aim of this study was to investigate the epidemic characteristics of *PEDV* that was circulating in Guangdong province, one of China’s major pig producing provinces. Clinical samples were collected from eight pig farms in Guangdong province between 2018 and 2019 and tested for the major porcine enteric pathogens, including *PEDV*, transmissible gastroenteritis virus (*TGEV)*, Swine enteric coronavirus (*SeCoV)*, Swine acute diarrhea syndrome coronavirus (*SADS-CoV)*, porcine deltacoronavirus (*PDCoV*), and porcine rotavirus (*RV*). As a result, only *PEDV* and *RV* were detected at a rate of 47.0% (16/34) and 18.6% (8/34), respectively. Coinfectoin with *PEDV* and RV occurred at a rate of *PEDV* 12.5% (2/16). Subsequently, the full-length S gene sequences of 13 *PEDV* strains were obtained, and phylogenetic analysis suggested the presence of GII-c group PEDV strains in this region (non-S-INDEL). Two novel common amino acid insertions (^55^T/IG^56^ and 551L) and one novel glycosylation site (1199^G+^) were detected when the CV777 and ZJ08 vaccine strains were compared. Furthermore, intragroup recombination events in the *S* gene regions 51–548 and 2478–4208 were observed in the *PEDV* strains studied. In summary, the observations provide current information on the incidence of viral agents causing swine diarrhea in southern China and detailed the genetic characteristics and evolutionary history of the dominant *PEDV* field strains. Our findings will aid in the development of an updated vaccine for the prevention and control of *PEDV* variant strains.

## Introduction

Porcine epidemic diarrhea, which is characterized by vomiting, acute watery diarrhea, dehydration, and weight loss in pigs, is a highly contagious and acute infectious disease caused by the porcine epidemic diarrhea virus (*PEDV)*. *PEDV* can infect pigs of any age, but particularly suckling piglets, and has a high mortality rate of up to 100% [1, 2]. Although the first *PEDV* outbreak was reported in 1971 in the United Kingdom, the *PEDV* cv777 strain was not isolated until 1977 in Belgium [1, 3, 4]. In the 1980s and 1990s, *PEDV* spread throughout many pig-producing countries in Europe and Asia, causing significant economic losses [5]. Since its discovery in China in the 1980s, *PEDV* has caused sporadic diarrhea outbreaks in Asian pig herds, causing even greater economic losses than in Europe [1, 6–10]. Except for an epidemic outbreak in northern Italy in 2005, only sporadic outbreaks of *PEDV* were reported in Europe [7]. Until 2010, a remarkable increase in *PEDV* outbreaks was reported in China’s pig-producing provinces [11]. A mutant strain was identified in which infected piglets defecate yellow watery stools, lose weight, and eventually die of dehydration, with a high mortality rate of up to 80%–100% in suckling piglets [12].

*PEDV* was first detected in the United States of America in 2013 and quickly spread throughout the country and into Europe [12–14]. Since then, a larger-scale epidemic outbreak of *PEDV* has impacted pig farms in China, Japan, and Korea, as well as countries in central and Eastern Europe, resulting in significant economic losses for the pig industry.

*PEDV* is a member of the *Coronaviridae* family’s *Alphacoronavirus* genus and the *Orthocoronavirinae* subfamily, which are enveloped viruses with an unsegmented positive sense RNA genome. The single-strand RNA genome of *PEDV* has a cap structure at 5’ and the poly (A) tail at the 3 ’end, with a size of approximately 30 kb. Four of the six open reading frames of the *PEDV* genome encode structural protein Spike (S), envelope (E), membrane (M), and nucleocapsid (N). The remaining 2/3 region of the 5 ’-terminal of ORF1a/1b and ORF3 encodes two viral RNA polymerase complex proteins. The S, E, M, and N structural proteins are located at the downstream of ORF1a and ORF1b [15]. ORF3 is an accessory gene that encodes a helper protein that aids in viral genome replication and translation [16]. The *PEDV* E glycoprotein is a component of the viral envelope that is involved in the formation and budding of the viral envelope [17]. The M protein contains 226 amino acids and has a molecular weight of 27–32 kDa. It is made up of two extracapsular parts, a trimer, and a carboxyl terminal within the virus to form a three-dimensional conformation [18]. The N protein is a phosphorylated basic protein with three functional domains that are all conserved: the N-terminal functional domain, the intermediate RNA binding domain, and the C-terminal binding domain. N protein is involved in the formation of viral sacs and is essential for coronavirus replication and transcription [19]. *PEDV* S protein, a type I fibrinoprotein, is involved in receptor binding, inducing neutralization antibodies, and membrane fusion. *PEDV* S protein consists of a signal peptide, an extracellular domain, a transmembrane region, and a cytoplasmic domain [20]. Cell proteases can splice S protein into S1 and S2, and the N-terminal region of S1 can bind to host cell receptors and mediate virus entry. In comparison to S1, the S2 region is more conserved, and it can mediate virus-cell membrane fusion and then internalization into host cells [21]. Remarkably, the S gene of *PEDV* contains the most variable regions in the entire *PEDV* genome and has the highest degree of diversity among all genes. Thus, the S gene has been used as a phylogenetic marker.

The diversity of the S gene is important in *PEDV* surveillance research because it provides a solid foundation for vaccine development, as well as disease prevention and control. A number of variant *PEDV* strains with insertions and deletions (INDEL) in the S gene were reported in the United States in 2014, altering viral antigenicity and pathogenicity [22, 23]. Although several studies on the genetic characterization and prevalence of *PEDV* have been conducted in central [24] and western China [25] prior to 2018, there has been little research on the prevalence of *PEDV* in southern China in recent years. The purpose of this study was to determine the viral agent prevalence in piglets suffering from acute diarrhea in Guangdong, China, and to provide updated information for genetic characterization of *PEDV* field strains.

## Materials and methods

### Ethics statement

This study was approved by the Research Ethics Committee of the College of Life Science and Engineering, Foshan University. Written informed consent was obtained from all owners whose animals were used in the study.

### Sample collection and cDNA synthesis

During 2018–2019, 19 feces and 15 fecal swabs were collected from diarrheal pigs in eight Guangdong province swine farms (Table 1). The pigs did not receive vaccines against *PEDV*, *TGEV* or *RV*. The samples were suspended in phosphate-buffered saline (PBS, pH = 7.4) and then clarified for 10 min at 4,000 rpm using centrifugation. The viral RNA was extracted using the Body Fluid Viral DNA/RNA Miniprep kit (Axygen, China) as directed by the manufacturer. Maxima H Minus First Strand cDNA Synthesis Kit was used to reverse transcribe viral RNA (Thermo Fisher Scientific, USA).

**Table 1:** Details of sample information in this study.

Swine farms N0.	Surveillance city	Collection date	Number of samples	*PEDV* positive	RV positive
1	Shixing	November, 2018	3	3/3	0/3
2	Lechang	January, 2019	5	3/5	0/5
3	Wujiang	January, 2018	5	0/5	0/5
4	Zhenjiang	January, 2018	4	4/4	1/4
5	Renhua	November,2018	6	0/6	3/6
6	Ruyuan	November, 2018	3	0/3	1/3
7	Qujiang	November, 2018	3	1/3	2/3
8	Gaofang	January, 2018	5	5/5	1/5

### Virus detection

The presence of swine *alphacoronaviruses*, such as *PEDV*, Transmissible gastroenteritis virus (*TGEV*), Swine enteric coronavirus (*SeCoV*), and Swine acute diarrhea syndrome coronavirus (*SADS-CoV*), was tested using a universal primer (Swine Cov F: 5’-AAACTGGAAYTTCASMTGG-3’; Swine Cov R: 5’-ACATARWAAGCCCAWC) designed by this study. Furthermore, the porcine deltacoronavirus (*PDCoV*) was detected using a primer set described previously by Sun et al. [26]. RV VP6 F: 5’-GAAACGGAATAGCTCCACAAT-3’ and RV VP6 R: 5’-GAATAATCAAATCCAGCCACC-3’ are primers. The presence of porcine rotavirus was detected by targeting the VP6 gene with an expected size of 271 bp. Thermo Fisher Scientific’s Phusion Hot Start II High-Fidelity PCR Master Mix (Thermo Fisher Scientific, USA) was used to amplify the *M* gene of swine alphacoronavirus and the *S* gene of *PDCoV*, which have expected sizes of 547 bp and 1763 bp, respectively. Table 1 lists the primers used in this study. The PCR products were purified using the Gene JET Extraction Kit from Thermo Fisher Scientific (Thermo Fisher Scientific, USA) and then subcloned into the pMD-18T vector (Takara, Japan). Sanger sequencing (Sangon Biotech, China) was used to obtain the *M* gene sequences, which were then BLAST searched against the GenBank database.

### *PEDV S* gene sequencing

The M gene positive samples were subjected to obtain the full-length sequence of the S gene. Two primers sets (*PEDV*-S1F, 5’-ATGACGCCATTTGTGGTTTTTC-3’ *PEDV*-S1R, 5’-GCCAGACTGAGATGGGACG-3’; *PEDV*-S2F:5’-TGGCAGTATTGGCTACGTCC-3’ *PEDV*-S2 R:5’-TGACGACTGTGTCAATCGTGT-3’) (Table 2) based on the conserved region of *PEDV* genome were designed to amplify the full-length sequence of *S* gene of *PEDV*. The S gene of *PEDV* was amplified using the Phusion Hot Start II High-Fidelity PCR Master Mix (Thermo Fisher Scientific, USA*PEDV*). Pre-denaturation at 98 °C for 30 s, followed by 35 cycles of denaturation at 98 °C for 10 s, annealing at 53 °C for 30s, and extension at 72 °C for 2 min, followed by extension fully at 72 °C for 10 min. Table 2 lists the primers available. Gene JET Gel Extraction Kit (Thermo Fisher Scientific, USA) was used to purify the amplified products, which were then subcloned into the pMD-18T vector (Takara, Japan) and sequenced using the Sanger method (Sangon Biotech, China). The sequences were assembled using MEGA. 7(Version 7.0.26).

**Table 2:** List of primers used in this study.

Gene name	Primer sequence	Length(bp)	Target gene
*Swine-CoV-M*	F: AAACTGGAAYTTCASMTGG	654	M
	R: ACATARWAAGCCCAWCCAGT		
*PEDV-S1*	F: ATGACGCCATTTGTGGTTTTTC	2356	S1
	R: GCCAGACTGAGATGGGACG		
*PEDV-S2*	F: TGGCAGTATTGGCTACGTCC	1925	S2
	R: TGACGACTGTGTCAATCGTGT		
*PDCOV-S1*	F: ATGCAGAGAGCTCTATTGATTATGAC	1763	S1
	R: AACTTGCAAGTACTCCGTCTGAACG		
*PDCOV-S2*	F: ATTTTCTCTTTCCGTTCAGACGGAG	1750	S2
	R: CTACCATTCCTTAAACTTAAAGGACG		
*RVA-VP6*	F: GAAACGGAATAGCTCCACAAT	271	VP6
	R: GAATAATCAAATCCAGCCACC		

### Phylogenetic analysis

The *S* gene sequences of 98 *PEDV* representative strains (Table 3) were extracted from the GenBank database for phylogenetic analysis to understand the evolution of the most common *PEDV* strains in Guangdong, China. The majority of the reference sequences were found in Asia, Europe, and North America. The MAFFT (Multiple Alignment using Fast Fourier Transform) embedded in the UGENE software was used to align multiple sequences (Version 36.0). The nucleotide (nt) and amino acid (aa) homology of S genes among the 13 strains were calculated using the Geneious software (Version 11.0.9) after alignment. The phylogenetic trees of *S* genes were constructed using the maximum likelihood (ML) method with 1,000 bootstrap replicates in IQ-TREE (Version 1.6.12) based on representative *PEDV* strains deposited in the GenBank. The phylogenetic tree was further annotated by FigTree (Version 1.4.3).

**Table 3:** PDEV reference strains described in this study.

Virus strain	Countries	Time	GenBank accession no.	Virus strain	Countries	Time	GenBank accession no.
CV777	Beigium	1988	AF353511	USA Colorado	USA	2013	KF272920
LZC	China	2006	EF185992	USA Indiana 17,846	USA	2013	KF452323
CH/S	China	1986	JN547228	USA/IA/2013/19321	USA	2013	KM975738
BJ-2011-1	China	2011	JN825712	USA/low107/2013	USA	2013	KJ645696
CHGD-01	China	2011	JN980698	USA/Minnesota52/2013	USA	2013	KJ645704
ZJCZ4	China	2011	JX524137	OH851	USA	2014	KJ399978
GD-1	China	2011	JX647847	OH1414	USA	2014	KJ408801
AJ1102	China	2012	JX188454	PC21A	USA	2014	KR078299
CH/GDGZ	China	2012	KF384500	FJzz1	China	2011	MK288006
GD-A	China	2012	JX112709	MH748550	China	2019	MH748550
AH2012	China	2012	KC210145	LZC	China	2007	EF185992
CH/YNKM-8	China	2013	KF761675	GD-B	China	2012	JX088695
K*PEDV*-9	Korea	1997	KF898124	IA2	USA	2013	KF468754
DR13	Korea	1999	DQ862099	SM98	Korea	2011	GU937797
attenuated-DR13	Korea	2002	JQ023162	YN144	China	2015	KT021232
KNU-0802	Korea	2008	GU180143	JS-HZ2012	China	2013	KC210147
KNU-0902	Korea	2009	GU180145	MEX/124/2014	USA	2015	KJ645700
CNU-091222-01	Korea	2009	JN184634	MMN	USA	2013	KF468752
KNU-1303	Korea	2013	KJ451038	PC21A	USA	2015	KR078299
K13JA12	Korea	2013	KJ539151	USA/Colorado/2013	USA	2013	KF272920
KNU-1401	Korea	2014	KJ451047	CH/JX-2/2013	China	2015	KJ526096
KNU-1402	Korea	2014	KJ451048	CH/JX-1/2013	China	2015	KF760557
K14JB01	Korea	2014	KJ539154	CH/HNAY/2015	China	2015	KR809885
IA1 USA	USA	2013	KF468753	LC	China	2012	JX489155
FL2013	China	2015	KP765609	CH/JXJA/2017	China	2018	MF375374
JS2008	China	2013	KC109141	CH/SCZY44/2017	China	2018	MH061338
*PEDV*-1556-Valencia-Requena	Spain	2020	MN692763	Hawaii/39249/2014	USA	2015	KP688354
*PEDV*_1611_Murcia_Lorca	Spain	2020	MN692768	EAS1	Thailand	2014	KR610991
SLOreBAS-2/2015	Slovenia	2016	KY019624	AVCT12	Thailand	2010	LC053455
SLO/JH-11/2015	Slovenia	2016	KU297956	ZJU/G1/2013	China	2013	KU664503
SNJ-P	China	2019	MK702008	85–7_China	China	2013	KX839246
LW/L	China	2019	MK392335	SC1402	China	2014	KP162057
OKN-1/JPN/2013	Japan	2015	LC063836	PPC-14	Korea	2014	MG781192
MYG-1/JPN/2014	Japan	2015	LC063838	SQ2014	China	2014	KP728470
EAS2	Thailand	2015	KR610992	SD-M	China	2012	JX560761
*PEDV*_1613_Murcia_Fuentealamo	Spain	2020	MN692769	YN15	China	2013	KT021228
*PEDV*_GER_L01014-K01_15–04_2015	Germany	2018	LT898420	YN1	China	2013	KT021227
*PEDV*_GER_L00906-K16_14–01_2014	Germany	2018	LT898430	VN/VAP1113	Vietnam	2013	KJ960179
*PEDV*_GER_L01020-K01_15–10_2015	Germany	2018	LT898413	YN30	China	2013	KT021229
HUA-14PED96	Viet Nam	2016	KT941120	CBR1	Thailand	2014	KR610993
SCDY523	China	2018	MH593144	IWT1	Japan	2014	LC063834
CHM	China	2013	KM887144	KB2013-4	China	2013	KX580953
CV777	China	2016	KT323979	SHQP/YM/2013	China	2013	KJ196348
JSLS-1/2015	China	2016	KX534205	CH/HNLH/2015	China	2015	KT199103
GDS01	China	2015	KM089829	NW8	China	2015	MF782687
CH/SCCD/2014	China	2017	KU975389	YC	China	2014	KU252649
CH/SCZG/2017	China	2018	MH061337	CH/GX/2015/750A	China	2015	KY793536
15V010/BEL/2015	Belgium	2015	KR003452	GER/L00719/2014	Germany	2014	LM645058
L00721/GER/2014	Germany	2014	LM645057	FR/001/2014	France	2014	KR011756

### Recombination analysis and N-linked glycosylation prediction

The evolution of coronaviruses, including *PEDV*, was aided by genome recombination. The recombination detection program (RDP v5) was used to determine the recombination event in the *PEDV* S gene, which included ninedetection algorithms (RDP, GENECONV, Bootscan, Maxchi, Chimaera, SiSscan, PhylPro, LARD, and 3Seq) [27]. The detection of potential recombinants was done using a *P*<0.01 threshold. As previously stated, N-linked glycosylation was predicted [28].

## Results

### Sample screening and sequencing

Sixteen of the thirty-four field samples (47.0%) were found to be positive for *PEDV*. In the fecal samples or fecal swabs, the TGEV and PDCoV were not found. From fecal samples and fecal swabs, seven and nine *PEDV* positive samples were identified, respectively. Based on the RT-PCR results, eight samples (23.5%) were tested to be porcine rotavirus (RV) positive. The co-infection rate of *PEDV* and RV was 12.5% (2/16) (Table 1). The complete S gene and a portion of the *M* gene were amplified and sequenced using Sanger sequencing. GDsg01-GDsg13 are the names of the 13 S genes that were discovered. The *S* genes from the five swine farms ranged in length from 4158 to 4164 nucleotides (nt), with nt and amino acid (aa) homology of 97.09%-99.95% and 96.77% -99.79%, respectively (S1 Fig). The sequences were determined using Sanger sequencing and deposited in GenBank with accession No. MW478760-MW478772 respectively.

### Phylogenetic analysis of *S* gene

**Fig 1:**
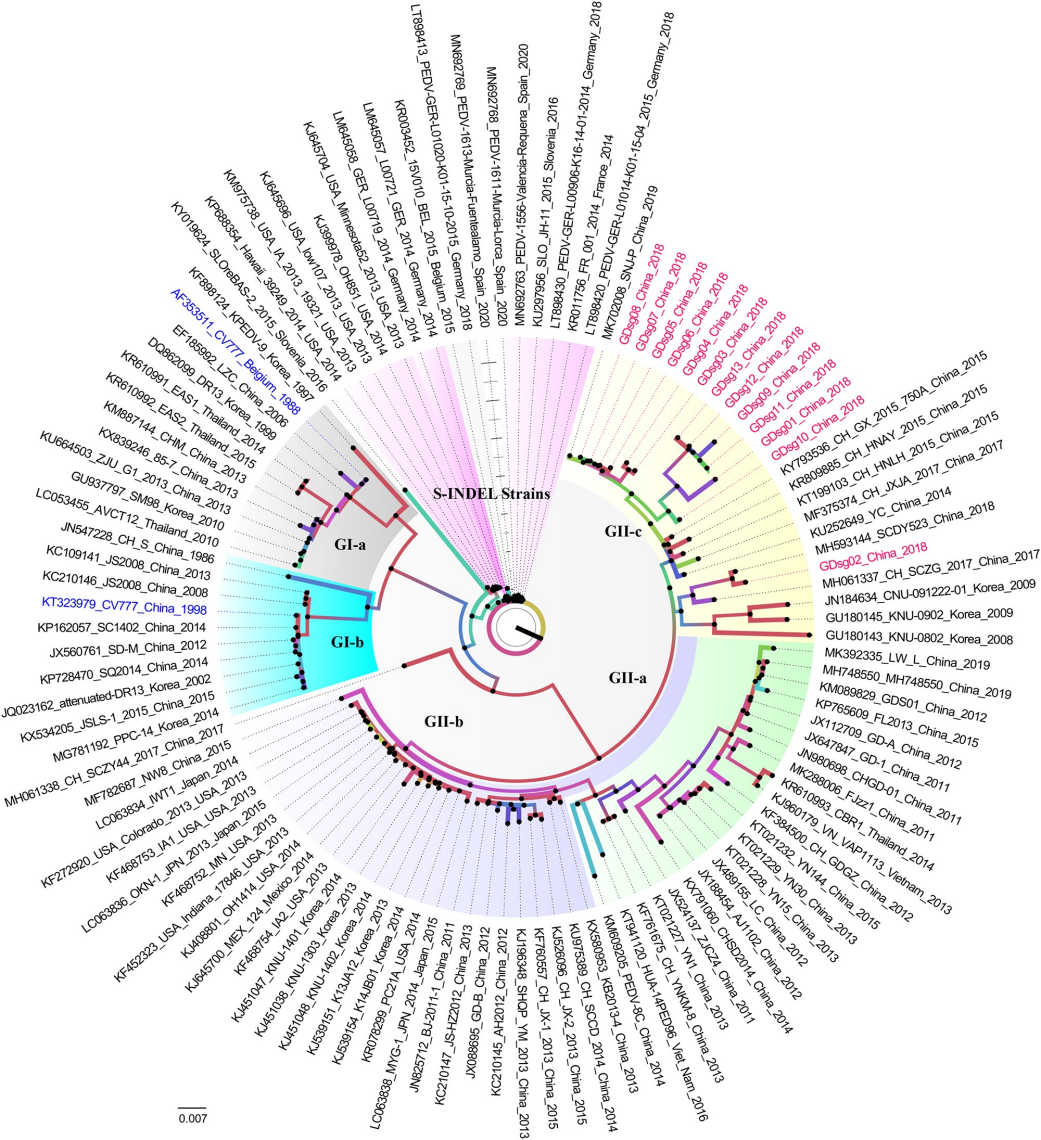
Phylogenetic analysis of full-length *S* gene of 13 *PEDV* strains collected in this study. MAFFT (Multiple Alignment using Fast Fourier Transform) in the UGENE software was used to align 98 *PEDV* reference strains with 13 *PEDV* strains (Version 36.0). With IQ-TREE, the phylogenetic tree was built using the maximum likelihood (ML) method with 1,000 bootstrap replicates (Version 1.6.12). *PEDV*’s *S* gene *PEDV* was divided into six categories: GI-a (light grey), GI-b (blue), GII-a (light green), GII-b (light cyan), and GII-c (light cyan) (light yellow). The CV777 reference strains are highlighted in blue, while the 13 strains reported in this study are highlighted in red. Nucleotide substitutions per site are indicated by a 0.007 bar.

A phylogenetic tree was constructed using full-length S genes from the 98 reference *PEDV* strains available in GenBank, and the 13 S-gene sequences obtained in this study to understand the phylogenetic relationship of these *PEDV* strains. *PEDV* strains were divided into two categories: traditional G1 and variant G2. The CV777 and SM98 strains were in the G1-a group. The attenuated vaccine strains were found in the G1-b group (CV777 and DR13). The 13 *PEDV* strains reported in this study had a strong association with the GII-c subgroup, according to our phylogenetic analysis (Fig 1).

### Sequence comparative analysis of S gene

**Fig 2:**
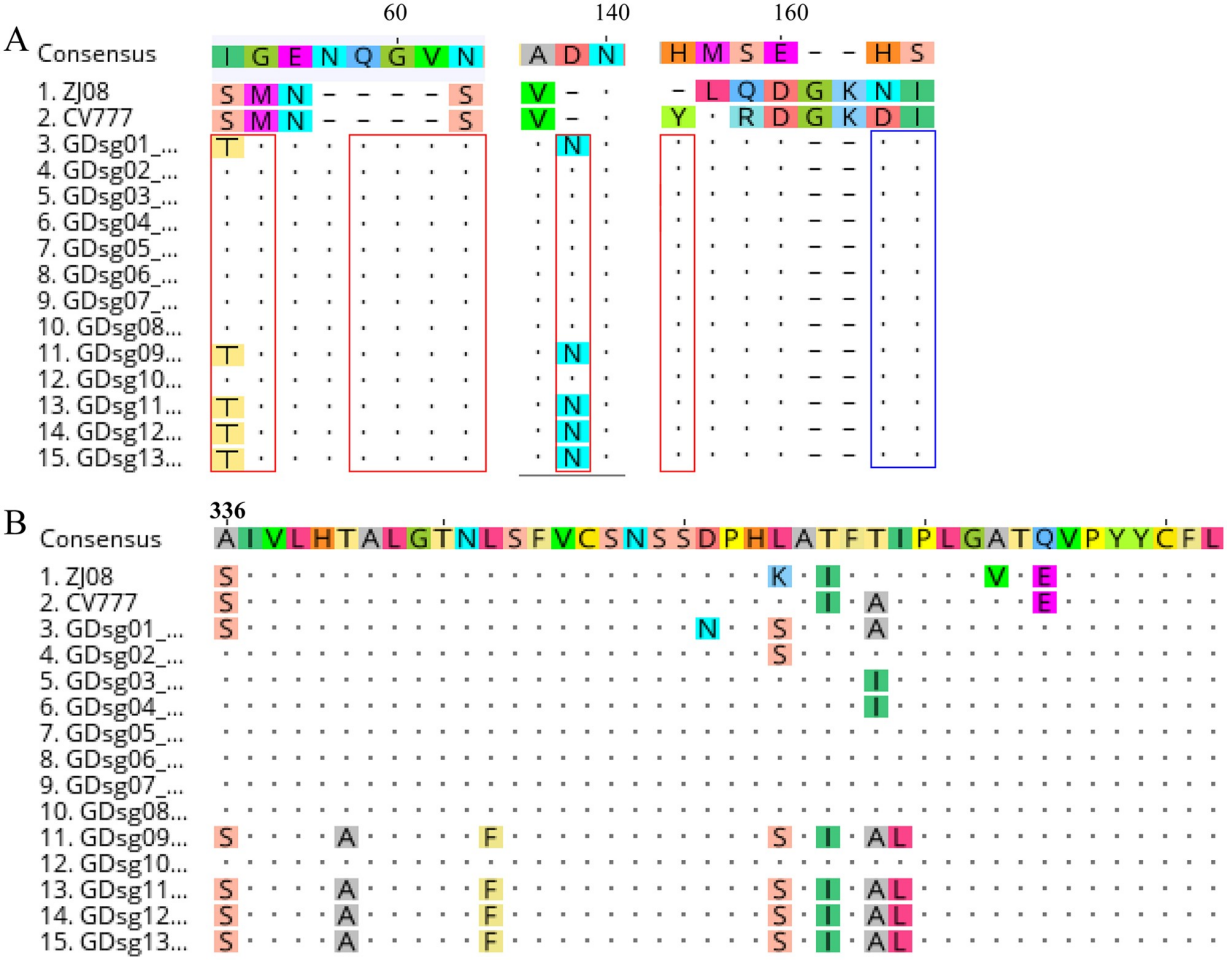
Analysis of animo acid mutations in the S protein of 13 *PEDV* strains. MUSCLE was used to align the sequences, and Geneious software (Version 11.0.9) was used to visualize them. (A) The common insertions (red box) and deletions (blue box) of amino acid (aa) mutations compared with the reference strains ZJ08 and CV777. (B). The regions in the S1 protein with a relative high polymorphism of mutations. (C) The predicted N-linked glycosylation sites of reference strain (CV777 and ZJ08), and 13 *PEDV* strains collected in this study. MUSCLE and the Geneious software were used to align the sequences (Version 11.0.9). The purple arrow represents the predicted N-linked glycosylation site based on the consensus N-X-S/T (X can be any amino acid except proline) glycosylation motif.

The *S* gene sequences were compared with classic strains and vaccine strains to further investigate the genetic characteristics of the 13 detected strains. As a result, the 13 *PEDV* strains shared homology with CV777 (KT323979), ZJ08, and AJ1102 (JX188454) of 93.4–93.8%, 93.3–93.6%, and 96.9–97.8%, respectively (Table 4). Compared with ZJ08, a total of 104 aa mutations were observed in the 13 *PEDV* strains of this study. S1 proteins exhibited 72.1% (75/104) of aa mutations and were majorly distributed in the S1-NTD and S1-CTD domains. The 13 strains shared a common aa deletion (^163^DI^164^) and five common aa insertions (^55^T/IG^56^, ^59^QGVN^62^, ^136^N, ^153^H, and ^551^L) (Fig 2A) compared to the reference strains CV777 and ZJ08. In addition, a high polymorphism (>30% in a specific aa position) of mutations (N = 19) such as ^62^Y→^66^H/Y, ^135^N→^135^N/D was observed in the S1 protein, especially the S1-CTD region (Fig 2B). In addition, two novel common mutations, including A520S and G612V, were identified in this study. The detailed list of aa mutations in the S protein of the 13 *PEDV* strains in comparison with vaccine strain ZJ08 is shown in Table 5.

**Table 4:** Sequence comparison of S gene of 13 *PEDV* strains and 3 vaccine strains.

*PEDV* strains	Percentage of nucleotide identity (%)		
	CV777 (KT323979)	ZJ08	AJ1102 (JX188454)
GDsg01 China 2018	93.4	93.3	97.2
GDsg02 China 2018	93.6	93.4	97.8
GDsg03 China 2018	93.7	93.5	97.3
GDsg04 China 2018	93.7	93.4	97.3
GDsg05 China 2018	93.8	93.6	97.6
GDsg06 China 2018	93.8	93.6	97.5
GDsg07 China 2018	93.8	93.6	97.6
GDsg08 China 2018	93.8	93.6	97.6
GDsg09 China 2018	93.7	93.5	97.0
GDsg10 China 2018	93.7	93.4	97.4
GDsg11 China 2018	93.7	93.5	96.9
GDsg12 China 2018	93.6	93.4	96.9
GDsg13 China 2018	93.7	93.4	96.9

**Table 5:** Statistics of mutations in the S protein of the 13 *PEDV* strains in comparison with ZJ08.

Domain	Mutations	Domain	Mutations	Domain	Mutations
SP	^2^TP^3^→^2^KS^3^		^231^S→^231^I/L		^763^L→^763^S
	^5^I→^5^T/N		^241^DS^242^→^241^EP/L^242^		^765^D→^765^S
	^15^L→^15^S	S1-CTD	^265^L/^265^V		^773^M→^773^T
S1-NTD	^27^QSTI^30^→^27^S/AANT^30^		^281^W→^281^L		^778^I→^783^M/I
	ΔΔ→^55^T/IG^56^		^283^I→^283^M	S2	^801^S→^801^S/T
	^56^MNS^58^→^56^GEN^58^		^298^MM^299^→^298^TI^299^		^805^V→^805^A
	ΔΔΔΔ→^59^QGVN^62^		^308^A→^308^V		^853^E→^853^E/D
	^60^S→^60^T		^323^F→^323^S		^890^G→^890^R
	^62^Y→^66^H/Y		^330^S→^330^S/A		^958^A→^958^V
	^64^GTGIE^68^→^64^AGQHP^68^		^335^T→^335^T/A		^962^L→^962^F
	^80^Y→^80^H		^341^L→^341^F/L		^964^T→^964^S
	^82^DS^83^→^82^RG^83^		^353^K→^353^S/L		^972^H→^972^Y
	^85^Q→^85^H		^355^I→^355^I/T		^994^L→^994^M/L
	^114^S→^114^N		^362^TI^363^→^362^AL/I^363^		^1027^N→^1027^K
	^116^I→^116^T		^362^V→^362^A		^1043^S→^1043^A
	^126^DN^127^→^126^NI^127^		^364^E→^364^Q		^1050^I→^1050^V
	^134^V→^134^A		^373^V→^373^L/V		^1095^A→^1095^A/S
	^135^N→^135^N/D		^377^K→^377^N		^1112^Q→^1112^Q/L
	Δ^136^N		^392^K→^392^R		^1139^E→^1134^E/D
			^429^D→^434^G/D		^1141^I→^1141^I/V
	Δ^153^H		^437^V→^442^I		^1161^N→^1161^N/D
	^154^LQDGK^158^→^154^MSEHS^158^		^473^S→^473^A		^1166^D→^1166^A
	^159^NI^160^→^159^ΔΔ^160^		^495^I→^495^I/T		^1172^GD^1173^→^1172^DE^1173^
	^173^A→^173^S		^516^A→^521^S/A		^1192^TY^1193^→^1192^NH^1193^
	^181^I→^181^F		Δ^551^L		^1231^S→^1231^R
	^191^R→^191^K		^600^G→^600^S		^1259^I→^1259^T
	^195^KRS^197^→^195^SGG^197^		^608^G→^613^V/G		^1261^P→^1261^S/P
	^205^T→^210^E		^632^Q→^632^E	TM	^1275^L→^1275^L/T
	^222^Y→^222^S	S1 other domain	^718^N→^718^N/S	Cyto	^1297^R→^1297^Q
	^224^E→^224^Q		^723^N→^723^S		^1358^G→^1358^C/G
					^1375^A→^1375^V

### *S* gene recombination analysis

**Fig 3:**
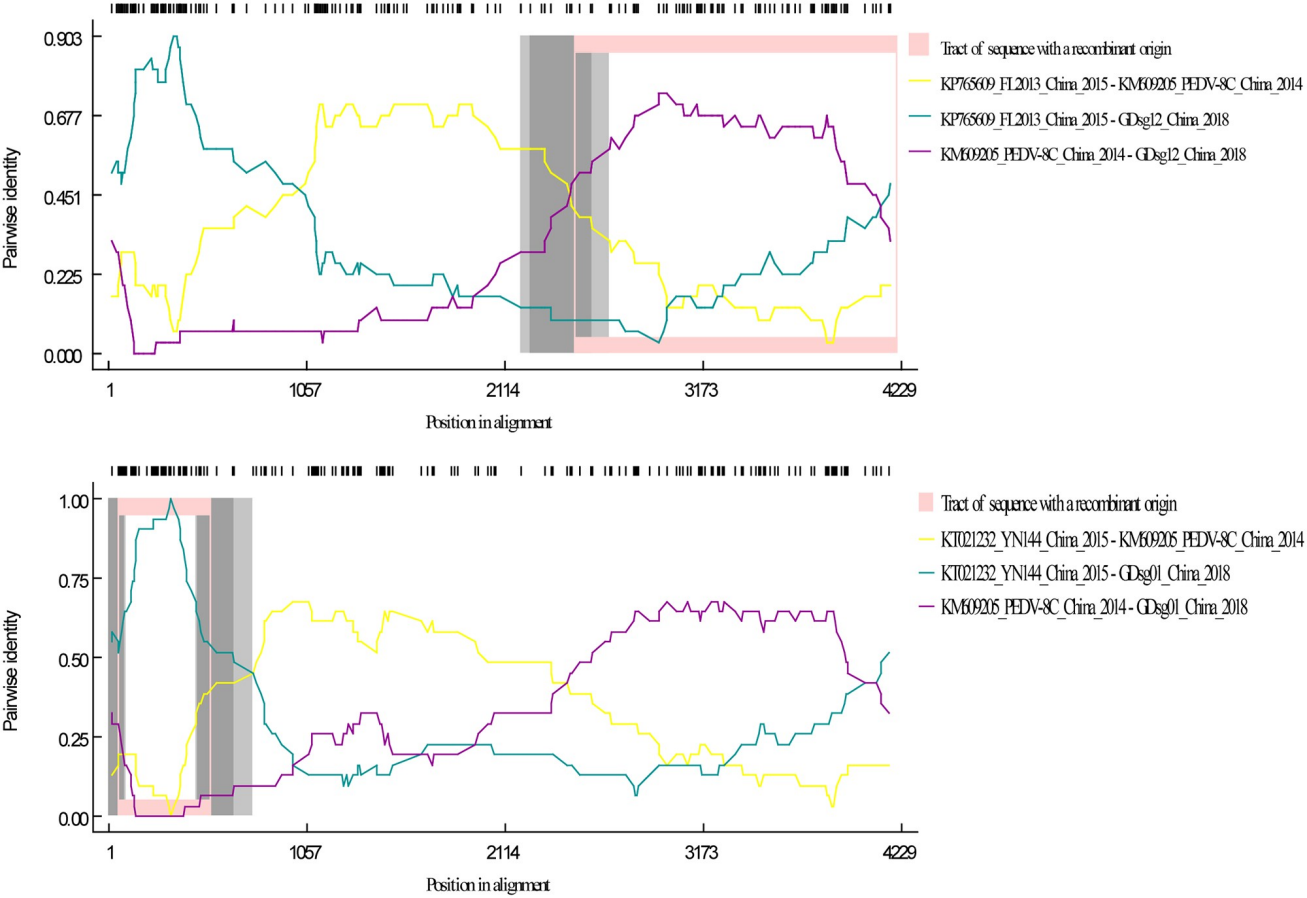
Detection of possible recombination events in the *PEDV* strains. The recombination detection software (RDP v5) was used to recognize recombination events in the GDsg12 (A) and GDsg01 (B) genomes using nine detection algorithms (RDP, GENECONV, Bootscan, Maxchi, Chimaera, SiSscan, PhylPro, LARD, 3Seq). The Y-axis represents the pairwise identity between the recombinant and its putative parents. The X-axis represents the position in alignment with a 30-nt sliding window. The comparison of recombinant-major parent, recombinant-minor parent, major-minor parent was indicated as cyan, purple, and yellow lines, respectively.

We performed a recombination analysis based on the 13 *PEDV* strains collected in this study as well as the 98 reference strains described above to understand the recombination events that occurred during the evolution of the *PEDV* strains circulating in Guangdong, China. The position 2478–4208 of the *S* gene of GDsg12 strain was predicted as a recombinant between KP765609 (major parent, GII-a) and KM609205 (minor parent, GII-a) (Fig 3A), which was supported by 6 detection methods (RDP, *P*-values ≤ 1.17× 10^−6^; Bootscan, *P*-values ≤ 1.08 ×10^−7^; Maxchi *P*-values ≤ 5.43×10^−9^; Chimaera, *P*-values ≤ 7.38×10^−9^; SiSscan, *P*-values ≤ 2.82×10^−8^; 3Seq, *P*-values ≤ 3.67×10^−10^). Similarly, 6 detection methods indicated that position 51–548 of the S gene of the GDsg01 strain was likely produced by intragroup recombination between KT021232 (major parent, GII-a) and KM609205 (minor parent, GII-a) (Fig 3B) (RDP, *P*-values ≤ 2.17×10^−11^; GENECONV, *P*-values ≤ 2.24×10^−9^; Bootscan, *P*-values ≤ 1.76 10^−11^; Maxchi, Chimaera, *P*-values ≤ 2.17×10^−8^; 3Seq, *P*-values ≤ 2.86×10^−6^) with a high degree of reliability.

### N-linked glycosylation prediction

**Fig 4:**
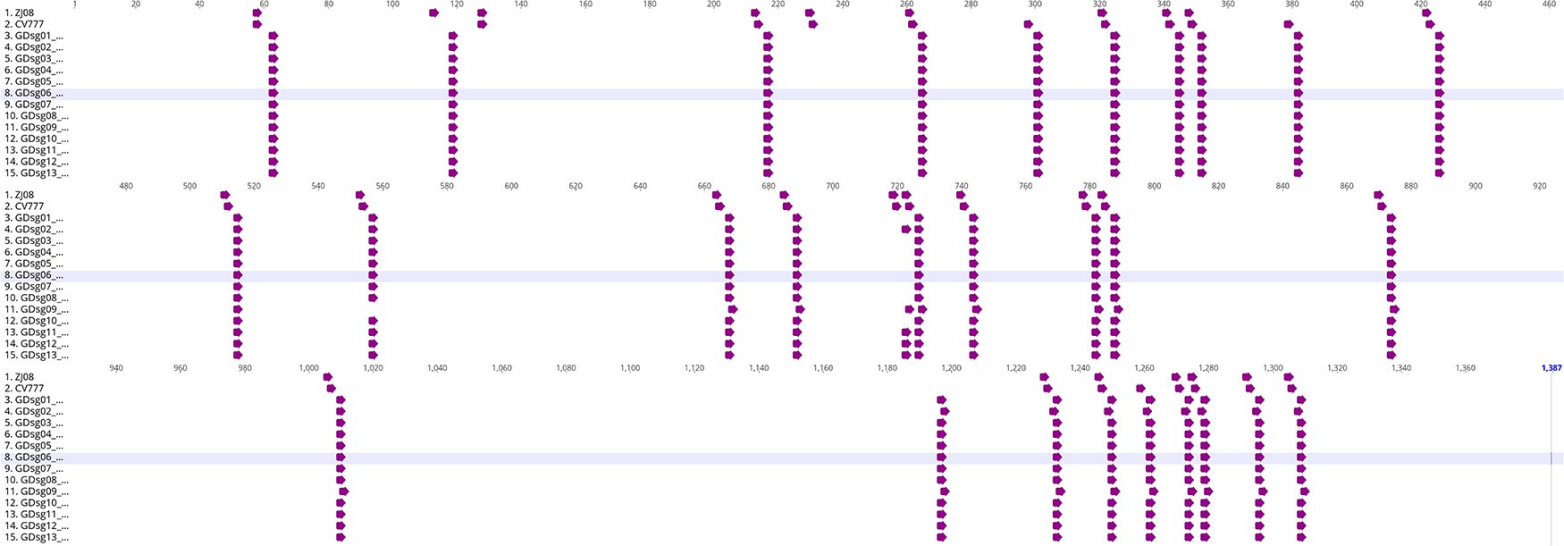
Predicted N-linked glycosylation sites of reference strains (CV777 and ZJ08) and 13 *PEDV* strains collected in this study. The sequences were aligned using MUSCLE with the Geneious software (Version 11.0.9). The purple arrow represents the predicted N-linked glycosylation site based on the consensus N-X-S/T (X can be any amino acid except proline) glycosylation motif.

The N-linked glycosylation sites were predicted based on the consensus N-X-S/T glycosylation motif. As a result, the 13 *PEDV* strains had 28–29 predicted N-glycosylation sites (G^+^) and showed a more similar pattern to CV777 than ZJ08 (Fig 4). Two (131^G-^, 235^G-^) and one common loss of glycosylation sites (235^G-^) were observed when compared with the reference strains ZJ08 and CV777, respectively. In contrast, the *PEDV* strains reported in this study gained three (302^G+^, 1199 ^G+^, and 1264 ^G+^) and one (1199^G+^) potential glycosylation sites compared with the ZJ08 and CV777, respectively. In addition, 61.5% (8/13) of *PEDV* strains reported in this study lost the glycosylation site at residue 725.

## Discussion

Several coronaviruses, including *PEDV*, *TGEV*, *SADS-CoV*, *SeCoV*, and *PDCoV*, have been identified in piglets suffering from acute diarrhea and vomiting [29–32]. CoV entry is mediated by the S glycoprotein, which is a critical factor in determining the virus’s tissue tropism and antigenicity. Additionally, the *S* gene of the coronavirus is prone to change through recombination or accumulation of point mutations, which can result in the virus losing its antigenicity and even the vaccine failing to work [33–35]. The molecular characterization of *PEDV*s is a major focus of swine diarrhea virus research due to the virus’s high prevalence.

During 2018–2019, we investigated the presence of swine diarrhea virus in eight pig farms in Guangdong, China, and then focused on the genetic characterization of *PEDV*. Our findings supported previous research [36], which indicated that *PEDV* is a leading cause of swine acute diarrhea in south China. We reported a detection rate of 47.0% for PDEV strains in fecal samples and fecal swabs collected from diarrheal pigs in eightswine farms. The reduction in TGEV reported in that study [36] is in agreement with our results. The gradual disappearance of TGEV has been attributed to the spread of porcine respiratory coronavirus, which conserved the majority of antigenic sites and caused a cross-protection against TGEV [37]. *PDCoV*, a newly identified member of the viral agents that cause swine diarrhea, was previously considered to be the second most prevalent swine diarrhea pathogen, following *PEDV* [38]. Sun et al. previously reported the genetic characterization of *PDCoV* in Shandong province during the same time period as this study and found a 50% co-infection rate of *PDEV* and *PDCoV* [26]. However, *PDCoV* was not detected in our study. Those observations indicated that the molecular epidemiology of the swine diarrhea virus varies greatly across China. Rotaviruses (RV), which belong to the Reoviridae family, have been identified as a major cause of viral gastroenteritis in young animals, such as piglets [39, 40]. We found an 18.6% detection rate for RV and a 12.5% co-infection rate for *PEDV* and RV. Surprisingly, a recent survey of pig farms in Brazil found porcine rotavirus B to be the primary viral agent (71.1%) in newborn piglets with acute viral gastroenteritis. In contrast, another study found that RT-PCR had a low detection rate of porcine RV (3%). The detection rate of porcine RV; however, varies depending on the detection method [41]. In this study, all *PEDV* stains were found to be clustered into GII-c (non-S-INDEL) subgroups. In a recent study, Tian et al. found that *PEDV* strains with the S gene of the GII-c subgroup were the most prevalent in Sichuan [25]. Furthermore, the nt identity of the PEDV strains in this study was similar to vaccine strains CV777 and ZJ08, but slightly higher than AJ1102 to. A genomic variation hotspot was also discovered in the S1-CTD region. It’s been suggested that the surface of *PEDV*’s S protein *PEDV* contains four major epitopes [42, 43]. Our findings revealed a number of novel common mutations, including A520S and G612V. These aa changes may have an effect on the virus’s antigenicity, leading to immune escape and vaccine failure. It’s worth noting that all 13 *PEDV* strains have the same newly discovered B-cell epitope (^722^SSTFNSTREL^731^) [42] of S protein *PEDV*. In addition to the previously reported common aa deletion (^163^DI^164^) and aa insertions (^59^QGVN^62^, ^136^N, and ^153^H), we report two new common aa insertions for *PEDV* strains circulating in south China: ^55^T/IG^56^ and ^551^L. Nevertheless, more research is needed into the impact of those common mutations on virus characterization, such as antigenicity and pathogenicity.

In addition to the accumulation of point mutations, homologous recombination among members of the same genus is a common way for the genetic evolution of coronaviruses. The recent SARS-CoV-2 outbreak was thought to be the result of cross-species recombination between bat and pangolin coronaviruses [44, 45]. Similarly, *PEDV* is intensively recombined between other members of the *Alphacoronavirus*, such as TGEV. In 2016, Italy [37], Germany [46] and Slovakia [47] reported the discovery of a novel swine enteric coronavirus with a backbone derived from *TGEV* and the S gene derived from *PEDV* [47]. Notably, a recent retrospective study indicated that the recombinant *SeCoV* circulating in Spain may have been misidentified as *PEDV* using S-protein or *S*-gene assays [48]. Our studies indicated that recombination occurred in both the S1 and S2 regions of GII-c *PEDV* strains, as supported by at least six highly reliable methods. The recombination events could be a result of pigs being transported and traded frequently in China.

In conclusion, our findings revealed that *PEDV* and porcine RV were the two main viral agents responsible for the outbreak of diarrhea on swine farms in China’s Guangdong province. A number of novel mutations were discovered, including common insertions like ^55^T/IG^56^ and 5^51^L. In addition, when compared to the vaccine strain, one common loss of glycosylation site (235^G-^) was observed. Intragroup recombination events were discovered in the S gene of the *PEDV* strains studied. Our findings highlight the critical need for the development of novel vaccines to combat recent new *PEDV* variants.

## Supporting information

S1 Fig The nucleotide (A) and amino acid (B) homology of S gene among the 13 *PEDV* strains in this study. The sequence homology was calculated by the Geneious software (Version 11.0.9) after multiple sequence alignment.
(TIF)

## References

[1] Pensaert MB, de Bouck P. A new coronavirus-like particle associated with diarrhea in swine. Arch Virol. 1978;58(3):243–7. Epub 1978/01/01. doi: 10.1007/BF01317606 83132 PMC7086830

[2] Shibata I, Tsuda T, Mori M, Ono M, Sueyoshi M, Uruno K. Isolation of porcine epidemic diarrhea virus in porcine cell cultures and experimental infection of pigs of different ages. Vet Microbiol. 2000;72(3–4):173–82. Epub 2000/03/23. doi: 10.1016/s0378-1135(99)00199-6 . 10727829 PMC7117361

[3] Wood EN. An apparently new syndrome of porcine epidemic diarrhoea. Vet Rec. 1977;100(12):243–4. Epub 1977/03/19. 888300 10.1136/vr.100.12.243

[4] Chasey D, Cartwright SF. Virus-like particles associated with porcine epidemic diarrhoea. Res Vet Sci. 1978;25(2):255–6. Epub 1978/09/01. doi: 10.1016/S0034-5288(18)32994-1 . 103154 PMC7130664

[5] Song D, Park B. Porcine epidemic diarrhoea virus: a comprehensive review of molecular epidemiology, diagnosis, and vaccines. Virus Genes. 2012;44(2):167–75. Epub 2012/01/25. doi: 10.1007/s11262-012-0713-1 . 22270324 PMC7089188

[6] Kusanagi K, Kuwahara H, Katoh T, Nunoya T, Ishikawa Y, Samejima T, et al. Isolation and serial propagation of porcine epidemic diarrhea virus in cell cultures and partial characterization of the isolate. J Vet Med Sci. 1992;54(2):313–8. Epub 1992/04/01. doi: 10.1292/jvms.54.313 . 1318752

[7] Martelli P, Lavazza A, Nigrelli AD, Merialdi G, Alborali LG, Pensaert MB. Epidemic of diarrhoea caused by porcine epidemic diarrhoea virus in Italy. Vet Rec. 2008;162(10):307–10. Epub 2008/03/11. doi: 10.1136/vr.162.10.307 . 18326842

[8] Pijpers A, van Nieuwstadt AP, Terpstra C, Verheijden JH. Porcine epidemic diarrhoea virus as a cause of persistent diarrhoea in a herd of breeding and finishing pigs. Vet Rec. 1993;132(6):129–31. Epub 1993/02/06. 8383370 10.1136/vr.132.6.129

[9] Pospischil A, Hess RG, Bachmann PA. Light microscopy and ultrahistology of intestinal changes in pigs infected with epizootic diarrhoea virus (EVD): comparison with transmissible gastroenteritis (TGE) virus and porcine rotavirus infections. Zentralbl Veterinarmed B. 1981;28(7):564–77. Epub 1981/01/01. doi: 10.1111/j.1439-0450.1981.tb01774.x . 6277107 PMC7165649

[10] Ben Salem AN, Chupin Sergei A, Bjadovskaya Olga P, Andreeva Olga G, Mahjoub A, Prokhvatilova Larissa B. Multiplex nested RT-PCR for the detection of porcine enteric viruses. J Virol Methods. 2010;165(2):283–93. Epub 2010/02/23. doi: 10.1016/j.jviromet.2010.02.010 . 20170679 PMC7112813

[11] Wang D, Fang L, Xiao S. Porcine epidemic diarrhea in China. Virus Res. 2016;226:7–13. Epub 2016/06/05. doi: 10.1016/j.virusres.2016.05.026 . 27261169 PMC7114554

[12] Li W, Li H, Liu Y, Pan Y, Deng F, Song Y, et al. New variants of porcine epidemic diarrhea virus, China, 2011. Emerg Infect Dis. 2012;18(8):1350–3. Epub 2012/07/31. doi: 10.3201/eid1808.120002 . 22840964 PMC3414035

[13] Stadler J, Zoels S, Fux R, Hanke D, Pohlmann A, Blome S, et al. Emergence of porcine epidemic diarrhea virus in southern Germany. BMC Vet Res. 2015;11:142. Epub 2015/07/03. doi: 10.1186/s12917-015-0454-1 . 26135732 PMC4487554

[14] Stevenson GW, Hoang H, Schwartz KJ, Burrough ER, Sun D, Madson D, et al. Emergence of Porcine epidemic diarrhea virus in the United States: clinical signs, lesions, and viral genomic sequences. J Vet Diagn Invest. 2013;25(5):649–54. Epub 2013/08/22. doi: 10.1177/1040638713501675 . 23963154

[15] Brian DA, Baric RS. Coronavirus genome structure and replication. Curr Top Microbiol Immunol. 2005;287:1–30. Epub 2004/12/22. doi: 10.1007/3-540-26765-4_1 . 15609507 PMC7120446

[16] Tian Y, Yu Z, Cheng K, Liu Y, Huang J, Xin Y, et al. Molecular characterization and phylogenetic analysis of new variants of the porcine epidemic diarrhea virus in Gansu, China in 2012. Viruses. 2013;5(8):1991–2004. Epub 2013/08/21. doi: 10.3390/v5081991 . 23955500 PMC3761238

[17] Corse E, Machamer CE. Infectious bronchitis virus E protein is targeted to the Golgi complex and directs release of virus-like particles. J Virol. 2000;74(9):4319–26. Epub 2001/02/07. doi: 10.1128/jvi.74.9.4319-4326.2000 . 10756047 PMC111949

[18] Chen JF, Sun DB, Wang CB, Shi HY, Cui XC, Liu SW, et al. Molecular characterization and phylogenetic analysis of membrane protein genes of porcine epidemic diarrhea virus isolates in China. Virus Genes. 2008;36(2):355–64. Epub 2008/01/25. doi: 10.1007/s11262-007-0196-7 . 18214664 PMC7088904

[19] Shi D, Shi H, Sun D, Chen J, Zhang X, Wang X, et al. Nucleocapsid Interacts with NPM1 and Protects it from Proteolytic Cleavage, Enhancing Cell Survival, and is Involved in PEDV Growth. Sci Rep. 2017;7:39700. Epub 2017/01/04. doi: 10.1038/srep39700 . 28045037 PMC5206633

[20] Cao L, Ge X, Gao Y, Zarlenga DS, Wang K, Li X, et al. Putative phage-display epitopes of the porcine epidemic diarrhea virus S1 protein and their anti-viral activity. Virus Genes. 2015;51(2):217–24. Epub 2015/08/22. doi: 10.1007/s11262-015-1234-5 . 26292945 PMC7089464

[21] Chang SH, Bae JL, Kang TJ, Kim J, Chung GH, Lim CW, et al. Identification of the epitope region capable of inducing neutralizing antibodies against the porcine epidemic diarrhea virus. Mol Cells. 2002;14(2):295–9. Epub 2002/11/22. . 12442904

[22] Vlasova AN, Marthaler D, Wang Q, Culhane MR, Rossow KD, Rovira A, et al. Distinct characteristics and complex evolution of PEDV strains, North America, May 2013-February 2014. Emerg Infect Dis. 2014;20(10):1620–8. Epub 2014/10/04. doi: 10.3201/eid2010.140491 . 25279722 PMC4193278

[23] Wang L, Byrum B, Zhang Y. New variant of porcine epidemic diarrhea virus, United States, 2014. Emerg Infect Dis. 2014;20(5):917–9. Epub 2014/04/23. doi: 10.3201/eid2005.140195 . 24750580 PMC4012824

[24] Cui JT, Qiao H, Hou CY, Zheng HH, Li XS, Zheng LL, et al. Characteristics of the spike and ORF3 genes of porcine epidemic diarrhea virus in Henan and Shanxi provinces of China. Arch Virol. 2020;165(10):2323–33. Epub 2020/07/28. doi: 10.1007/s00705-020-04744-x . 32715325 PMC7382918

[25] Tian Y, Yang X, Li H, Ma B, Guan R, Yang J, et al. Molecular characterization of porcine epidemic diarrhea virus associated with outbreaks in southwest China during 2014–2018. Transbound Emerg Dis. 2020. Epub 2020/12/12. doi: 10.1111/tbed.13953 . 33306274

[26] Sun W, Wang L, Huang H, Wang W, Cao L, Zhang J, et al. Genetic characterization and phylogenetic analysis of porcine deltacoronavirus (PDCoV) in Shandong Province, China. Virus Res. 2020;278:197869. Epub 2020/01/22. doi: 10.1016/j.virusres.2020.197869 . 31962065 PMC7114949

[27] Martin DP, Murrell B, Golden M, Khoosal A, Muhire B. RDP4: Detection and analysis of recombination patterns in virus genomes. Virus Evol. 2015;1(1):vev003. Epub 2015/05/26. doi: 10.1093/ve/vev003 . 27774277 PMC5014473

[28] Wen F, Li W, Guo J, Yang J, Zhang X, Mei K, et al. Genetic characterization of a novel genotype H9N2 avian influenza virus from chicken in South China. J Infect. 2020;81(5):816–46. Epub 2020/09/22. doi: 10.1016/j.jinf.2020.09.011 . 32956732

[29] Fu X, Fang B, Liu Y, Cai M, Jun J, Ma J, et al. Newly emerged porcine enteric alphacoronavirus in southern China: Identification, origin and evolutionary history analysis. Infect Genet Evol. 2018;62:179–87. Epub 2018/04/29. doi: 10.1016/j.meegid.2018.04.031 . 29704627 PMC7106130

[30] Xu Z, Zhong H, Zhou Q, Du Y, Chen L, Zhang Y, et al. A Highly Pathogenic Strain of Porcine Deltacoronavirus Caused Watery Diarrhea in Newborn Piglets. Virol Sin. 2018;33(2):131–41. Epub 2018/03/24. doi: 10.1007/s12250-018-0003-8 . 29569144 PMC6178105

[31] Zhou L, Sun Y, Lan T, Wu R, Chen J, Wu Z, et al. Retrospective detection and phylogenetic analysis of swine acute diarrhoea syndrome coronavirus in pigs in southern China. Transbound Emerg Dis. 2019;66(2):687–95. Epub 2018/09/02. doi: 10.1111/tbed.13008 . 30171801 PMC7168530

[32] Zhou P, Fan H, Lan T, Yang XL, Shi WF, Zhang W, et al. Fatal swine acute diarrhoea syndrome caused by an HKU2-related coronavirus of bat origin. Nature. 2018;556(7700):255–8. Epub 2018/04/06. doi: 10.1038/s41586-018-0010-9 . 29618817 PMC7094983

[33] Chen N, Li S, Zhou R, Zhu M, He S, Ye M, et al. Two novel porcine epidemic diarrhea virus (PEDV) recombinants from a natural recombinant and distinct subtypes of PEDV variants. Virus Res. 2017;242:90–5. Epub 2017/09/28. doi: 10.1016/j.virusres.2017.09.013 . 28947336

[34] Lu Y, Su X, Du C, Mo L, Ke P, Wang R, et al. Genetic Diversity of Porcine Epidemic Diarrhea Virus With a Naturally Occurring Truncated ORF3 Gene Found in Guangxi, China. Front Vet Sci. 2020;7:435. Epub 2020/08/15. doi: 10.3389/fvets.2020.00435 . 32793651 PMC7393948

[35] Sun J, Li Q, Shao C, Ma Y, He H, Jiang S, et al. Isolation and characterization of Chinese porcine epidemic diarrhea virus with novel mutations and deletions in the S gene. Vet Microbiol. 2018;221:81–9. Epub 2018/07/10. doi: 10.1016/j.vetmic.2018.05.021 . 29981713 PMC7117340

[36] Zhao J, Shi BJ, Huang XG, Peng MY, Zhang XM, He DN, et al. A multiplex RT-PCR assay for rapid and differential diagnosis of four porcine diarrhea associated viruses in field samples from pig farms in East China from 2010 to 2012. J Virol Methods. 2013;194(1–2):107–12. Epub 2013/08/31. doi: 10.1016/j.jviromet.2013.08.008 . 23988656

[37] Boniotti MB, Papetti A, Lavazza A, Alborali G, Sozzi E, Chiapponi C, et al. Porcine Epidemic Diarrhea Virus and Discovery of a Recombinant Swine Enteric Coronavirus, Italy. Emerg Infect Dis. 2016;22(1):83–7. Epub 2015/12/23. doi: 10.3201/eid2201.150544 . 26689738 PMC4696687

[38] Song D, Zhou X, Peng Q, Chen Y, Zhang F, Huang T, et al. Newly Emerged Porcine Deltacoronavirus Associated With Diarrhoea in Swine in China: Identification, Prevalence and Full-Length Genome Sequence Analysis. Transbound Emerg Dis. 2015;62(6):575–80. Epub 2015/08/08. doi: 10.1111/tbed.12399 . 26250097 PMC7169704

[39] Martella V, Banyai K, Matthijnssens J, Buonavoglia C, Ciarlet M. Zoonotic aspects of rotaviruses. Vet Microbiol. 2010;140(3–4):246–55. Epub 2009/09/29. doi: 10.1016/j.vetmic.2009.08.028 . 19781872

[40] Molinari BL, Possatti F, Lorenzetti E, Alfieri AF, Alfieri AA. Unusual outbreak of post-weaning porcine diarrhea caused by single and mixed infections of rotavirus groups A, B, C, and H. Vet Microbiol. 2016;193:125–32. Epub 2016/09/08. doi: 10.1016/j.vetmic.2016.08.014 . 27599939

[41] Zhang F, Luo S, Gu J, Li Z, Li K, Yuan W, et al. Prevalence and phylogenetic analysis of porcine diarrhea associated viruses in southern China from 2012 to 2018. BMC Vet Res. 2019;15(1):470. Epub 2019/12/29. doi: 10.1186/s12917-019-2212-2 . 31881873 PMC6935106

[42] Kong N, Meng Q, Jiao Y, Wu Y, Zuo Y, Wang H, et al. Identification of a novel B-cell epitope in the spike protein of porcine epidemic diarrhea virus. Virol J. 2020;17(1):46. Epub 2020/04/05. doi: 10.1186/s12985-020-01305-1 . 32245493 PMC7119268

[43] Li RF, Qiao SL, Yang YY, Su YF, Zhao P, Zhou EM, et al. Phylogenetic analysis of porcine epidemic diarrhea virus (PEDV) field strains in central China based on the ORF3 gene and the main neutralization epitopes. Archives of Virology. 2014;159(5):1057–65. doi: 10.1007/s00705-013-1929-7 24292967 PMC7087087

[44] Li X, Giorgi EE, Marichannegowda MH, Foley B, Xiao C, Kong XP, et al. Emergence of SARS-CoV-2 through recombination and strong purifying selection. Sci Adv. 2020;6(27). Epub 2020/09/17. doi: 10.1126/sciadv.abb9153 . 32937441 PMC7458444

[45] Wen F, Yu H, Guo J, Li Y, Luo K, Huang S. Identification of the hyper-variable genomic hotspot for the novel coronavirus SARS-CoV-2. J Infect. 2020;80(6):671–93. Epub 2020/03/08. doi: 10.1016/j.jinf.2020.02.027 . 32145215 PMC7126159

[46] Akimkin V, Beer M, Blome S, Hanke D, Hoper D, Jenckel M, et al. New Chimeric Porcine Coronavirus in Swine Feces, Germany, 2012. Emerg Infect Dis. 2016;22(7):1314–5. Epub 2016/04/14. doi: 10.3201/eid2207.160179 . 27070291 PMC4918154

[47] Mandelik R, Sarvas M, Jackova A, Salamunova S, Novotny J, Vilcek S. First outbreak with chimeric swine enteric coronavirus (SeCoV) on pig farms in Slovakia—lessons to learn. Acta Vet Hung. 2018;66(3):488–92. Epub 2018/09/29. doi: 10.1556/004.2018.043 . 30264613

[48] de Nova PJG, Cortey M, Diaz I, Puente H, Rubio P, Martin M, et al. A retrospective study of porcine epidemic diarrhoea virus (PEDV) reveals the presence of swine enteric coronavirus (SeCoV) since 1993 and the recent introduction of a recombinant PEDV-SeCoV in Spain. Transbound Emerg Dis. 2020. Epub 2020/06/09. doi: 10.1111/tbed.13666 . 32511876

