# Supplementary figures and images for: Genetic characterization and phylogenetic analysis of porcine epidemic diarrhea virus in Guangdong, China, between 2018 and 2019
Source: PLoS One. 2021 Jun 24;16(6):e0253622. doi: 10.1371/journal.pone.0253622 (PMC8224968; doi:10.1371/journal.pone.0253622)

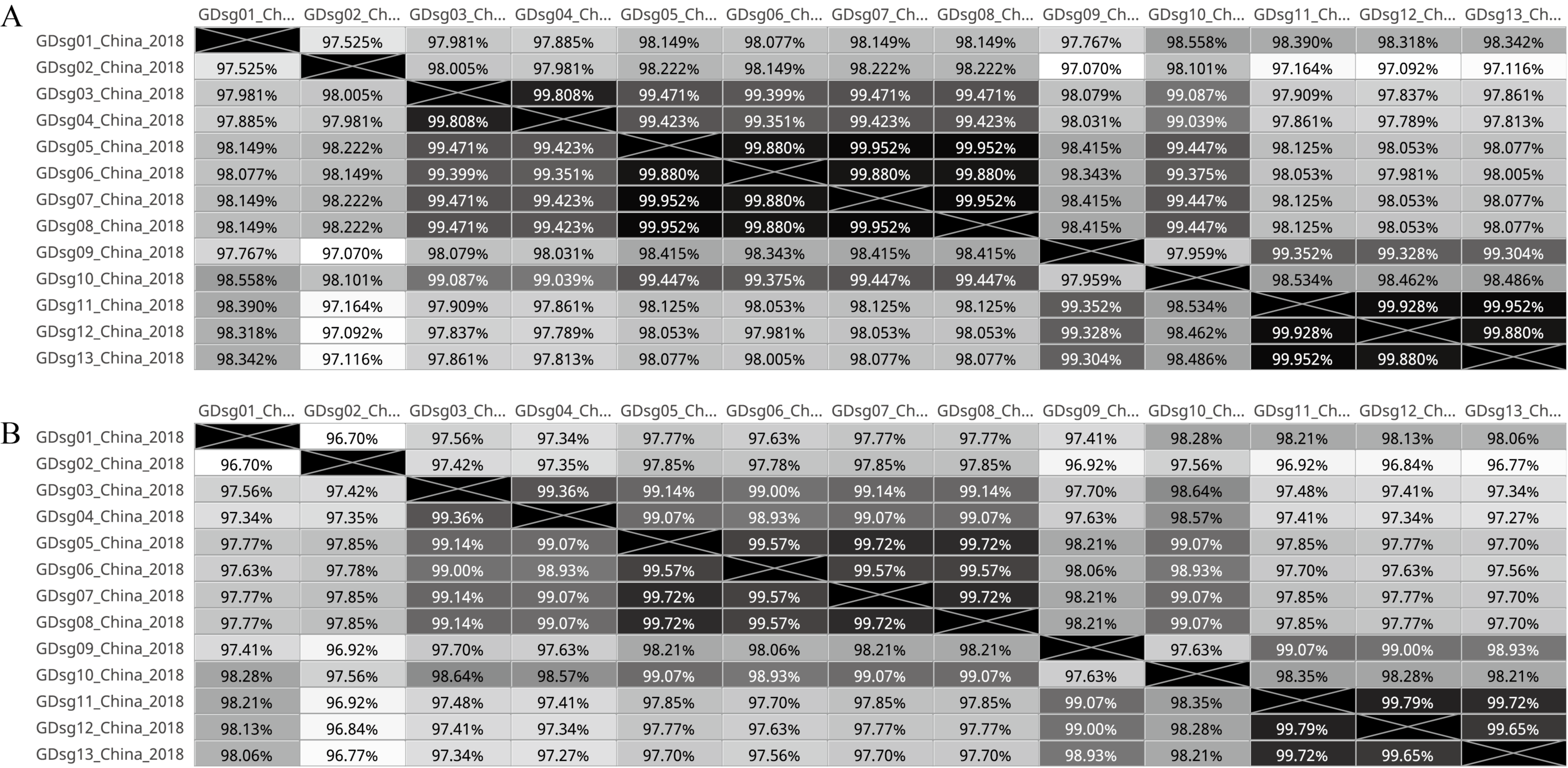

Supplement: S1 Fig — The sequence homology was calculated by the Geneious software (Version 11.0.9) after multiple sequence alignment. (TIF) [file pone.0253622.s001.tif]
